# Bis(1,3-dimethyl-1,3-diazinan-2-one)dinitratodioxidouranium(VI)

**DOI:** 10.1107/S1600536810049883

**Published:** 2010-12-04

**Authors:** Tomoya Suzuki, Takeshi Kawasaki, Yasuhisa Ikeda

**Affiliations:** a Research Laboratory for Nuclear Reactors, Tokyo Institute of Technology, 2-12-1-N1-34 Ookayama, Meguro-ku, Tokyo 152-8550, Japan

## Abstract

The title compound, [U(NO_3_)_2_O_2_(C_6_H_12_N_2_O)_2_], exhibits a hexa­gonal–bipyramidal geometry around the U^VI^ ion, which is situated on an inversion centre and coordinated by two oxide ligands in the axial positions, and four O atoms from two bidentate NO_3_^−^ and two O atoms from two 1,3-dimethyl-1,3-diazinan-2-one (DMPU) ligands in the equatorial plane. These ligands are located in *trans* positions. The –(CH_2_)_3_– moiety in the DMPU ligand is disordered over two positions in a 0.786 (11):0.214 (11) ratio.

## Related literature

For the structures of uran­yl(VI) nitrate complexes, see: Alcock *et al.* (1990 ▶); Cao *et al.* (1993 ▶, 1999 ▶); Ikeda *et al.* (2004 ▶); Kannan *et al.* (2008 ▶); Koshino *et al.* (2005 ▶); Pennington *et al.* (1988 ▶); Takao *et al.* (2008 ▶); van Vuuren & van Rooyen (1988 ▶); Varga *et al.* (2003 ▶); Villiers *et al.* (2004 ▶).
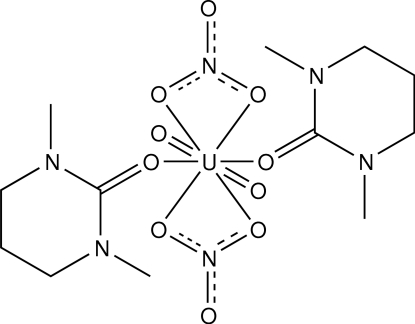


## Experimental

### 

#### Crystal data

[U(NO_3_)_2_O_2_(C_6_H_12_N_2_O)_2_]
*M* *_r_* = 650.40
Triclinic, 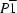

*a* = 7.8529 (6) Å
*b* = 8.7706 (6) Å
*c* = 9.1990 (6) Å
α = 115.611 (2)°
β = 113.348 (2)°
γ = 91.041 (2)°
*V* = 510.62 (6) Å^3^
*Z* = 1
Mo *K*α radiation
μ = 8.01 mm^−1^
*T* = 173 K
0.17 × 0.13 × 0.12 mm

#### Data collection

Rigaku R-AXIS RAPID diffractometer
Absorption correction: multi-scan (*ABSCOR*; Higashi, 1995 ▶) *T*_min_ = 0.343, *T*_max_ = 0.447
4800 measured reflections
2307 independent reflections
2306 reflections with *I* > 2σ(*I*)
*R*_int_ = 0.020

#### Refinement

*R*[*F* ^2^ > 2σ(*F* ^2^)] = 0.017
*wR*(*F* ^2^) = 0.045
*S* = 1.06
2307 reflections
143 parameters
H-atom parameters constrained
Δρ_max_ = 0.88 e Å^−3^
Δρ_min_ = −0.93 e Å^−3^

### 

Data collection: *PROCESS-AUTO* (Rigaku, 2006 ▶); cell refinement: *PROCESS-AUTO*; data reduction: *CrystalStructure* (Rigaku/MSC, 2006 ▶); program(s) used to solve structure: *SIR92* (Altomare *et al.*, 1994 ▶); program(s) used to refine structure: *SHELXL97* (Sheldrick, 2008 ▶); molecular graphics: *CrystalMaker* (*CrystalMaker*, 2007 ▶); software used to prepare material for publication: *CrystalStructure*.

## Supplementary Material

Crystal structure: contains datablocks global, I. DOI: 10.1107/S1600536810049883/kj2159sup1.cif

Structure factors: contains datablocks I. DOI: 10.1107/S1600536810049883/kj2159Isup2.hkl

Additional supplementary materials:  crystallographic information; 3D view; checkCIF report

## Figures and Tables

**Table 1:** Selected bond lengths (Å)

U1—O1	1.774 (2)
U1—O2	2.363 (2)
U1—O4	2.526 (2)
U1—O3	2.549 (2)

Symmetry code: (i) 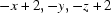.

**Fig. 1.**
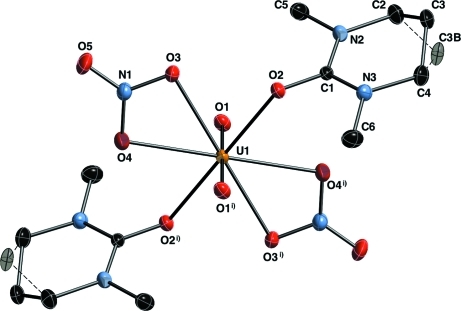
Molecular view of I with 30% thermal ellipsoids [symmetry code: i) -x + 2, -y, -z + 2]. Hydrogen atoms are omitted for clarity.

## supplementary crystallographic information

### Comment

Crystal structures of various uranyl(VI) nitrate complexes with neutral unidentate ligands (*L*) have been reported. The uranyl(VI) nitrate complexes normally have a conformation of UO_2_(NO_3_)_2_(*L*)_2_ (Cao *et al.*, 1999; Cao *et al.*, 1993; Ikeda *et al.*, 2004; Kannan *et al.*, 2008; Koshino *et al.*, 2005; Pennington *et al.*, 1988; Takao, *et al.*, 2008; van Vuuren & van Rooyen 1988; Varga *et al.*, 2003; Villiers *et al.*, 2004). The UO_2_(NO_3_)_2_(*L*)_2_ complexes exhibit hexagonal bipyramidal geometry, in which the U^VI^ atom is coordinated by two oxo ligands in the axial positions, and four oxygen atoms from two bidentate NO_3_^-^ and two donating atoms from two *L* in the equatorial plane. These ligands are located in the *trans* positions. Recently, we have reported that *N*-cyclohexyl-2-pyrrolidone (NCP) can selectively precipitate uranyl(VI) species in HNO_3_ aqueous solution and that the precipitate has an above typical molecular structure, *i.e.*, UO_2_(NO_3_)_2_(NCP)_2_ (Ikeda *et al.*, 2004; Varga *et al.*, 2003). Similarly, we have also studied other *N*-alkyl-2-pyrrolidone (NRP) (Ikeda *et al.*, 2004; Koshino *et al.*, 2005; Takao *et al.*, 2008; Varga *et al.*, 2003), 2-pyrrolidone(NHP) (Ikeda *et al.*, 2004) modification: 2-pyrrolidone(NHP) (Takao *et al.*, 2008), and 1,3-dimethyl-imidazolidone (DMI) (Koshino *et al.*, 2005). We report herein the synthesis and crystal characterization of the new uranyl(VI) complex UO_2_(NO_3_)_2_(DMPU)_2_ (I) (DMPU = 1,3-dimethyl-1,3-diazinan-2-one (*N*, *N*'-dimethylpropyleneurea)).

The molecular structure of the title complex is shown in Fig. 1. U1 has a hexagonal bipyramidal coordination geometry. The two uranyl oxo atoms (O1) from the uranyl(VI) ion occupy the axial position of U1, and two carbonyl oxygen atoms (O2) from the two unidentate DMPU and four oxygen atoms (two O3 and two O4) from the two bidentate NO_3_^-^ are situated in the *trans* positions in the equatorial plane of U1 (Fig. 1). The selected parameters are listed in Table 1. These structural features are similar to those of uranyl(VI) nitrate complexes with NRPs (Ikeda *et al.*, 2004; Koshino *et al.*, 2005; Takao *et al.*, 2008; Varga *et al.*, 2003), 2-imidazolidone type ligands [1,3-dibutyl-imidazolidone (DBI) and DMI modification: 1,3-dibutyl-imidazolium (DBI) (Cao *et al.*, 1999) and DMI (Koshino *et al.*, 2005)] and tetramethylurea (TMU) (van Vuuren & van Rooyen, 1988). The U—O_carbo_ bond length of the title complex is slightly shorter than those of uranyl(VI) nitrate complexes with NHP [2.414 (3) Å] (Takao *et al.*, 2008), *N*-cyclohexylmethyl-2- pyrrolidone [2.383 Å] (Koshino *et al.*, 2005), *N*-(1-ethylpropyl)-2-pyrrolidone [2.372 (2) Å] (Takao *et al.*, 2008), *N*-neopentyl-2-pyrrolidone [2.382 (3), 2.389 (3) Å] (Takao *et al.*, 2008), and NRPs having alkyl chains of carbon number 2 ~4 (about 2.37 ~2.4 Å) (Ikeda *et al.*, 2004; Koshino *et al.*, 2005; Takao *et al.*, 2008). On the other hand, The U—O_carbo_ bond of I is slightly longer than those of uranyl(VI) nitrate complexes with TMU [2.335 (3) Å] (van Vuuren & van Rooyen, 1988), urea [2.341 (5), 2,348 (5) Å] (Alcock *et al.*, 1990), DBI [2.345 (3) Å] (Cao *et al.*, 1999), and NCP [2.348 (2) Å] (Varga, *et al.*, 2003; Ikeda *et al.*, 2004). The differences in U—O bonds are considered to be due to those in donicity and size of *L*. In the dmpu ligand, C3 and C3B display disorder in a 0.786 (11) and 0.214 (11) occupancy ratio.

### Experimental

Uranyl stock solution of 0.5 *M* (*M* = mol dm^-3^) was prepared by dissolving UO_2_(NO_3_)_2_^.^6H_2_O in 3 *M* HNO_3_ aqueous solution. To 1 ml of the UO_2_^2+^ solution was added 1 mmol of DMPU with vigorous stirring. Yellow precipitate was obtained. The resulting precipitate was filtered off, and washed with hexane. The precipitate was recrystallized from dichloromethane.

### Refinement

All H atoms were positionated geometrically, with C—H 0.98 and 0.99 Å for methyl and methylene H atoms, and constrated to ride on their parent atoms, with *U*_iso_(H) = 1.2*U*_eq_(C).

### Crystal data

**Table:**

[U(NO_3_)_2_O_2_(C_6_H_12_N_2_O)_2_]	*Z* = 1
*M**_r_* = 650.40	*F*(000) = 310
Triclinic, *P*1	*D*_x_ = 2.115 Mg m^−^^3^
Hall symbol: -P 1	Mo *K*α radiation, λ = 0.71075 Å
*a* = 7.8529 (6) Å	Cell parameters from 6036 reflections
*b* = 8.7706 (6) Å	θ = 3.5–27.5°
*c* = 9.1990 (6) Å	µ = 8.01 mm^−^^1^
α = 115.611 (2)°	*T* = 173 K
β = 113.348 (2)°	Block, yellow
γ = 91.041 (2)°	0.17 × 0.13 × 0.12 mm
*V* = 510.62 (6) Å^3^	

### Data collection

**Table:**

Rigaku R-AXIS RAPID diffractometer	2307 independent reflections
Radiation source: fine-focus sealed tube	2306 reflections with *F*^2^ > 2σ(*F*^2^)
graphite	*R*_int_ = 0.020
Detector resolution: 10.00 pixels mm^-1^	θ_max_ = 27.5°, θ_min_ = 3.5°
ω scans	*h* = −10→10
Absorption correction: multi-scan (*ABSCOR*; Higashi, 1995)	*k* = −10→11
*T*_min_ = 0.343, *T*_max_ = 0.447	*l* = −11→11
4800 measured reflections	

### Refinement

**Table:**

Refinement on *F*^2^	Primary atom site location: structure-invariant direct methods
Least-squares matrix: full	Secondary atom site location: difference Fourier map
*R*[*F*^2^ > 2σ(*F*^2^)] = 0.017	Hydrogen site location: inferred from neighbouring sites
*wR*(*F*^2^) = 0.045	H-atom parameters constrained
*S* = 1.06	*w* = 1/[σ^2^(*F*_o_^2^) + (0.0297*P*)^2^ + 0.4772*P*] where *P* = (*F*_o_^2^ + 2*F*_c_^2^)/3
2307 reflections	(Δ/σ)_max_ < 0.001
143 parameters	Δρ_max_ = 0.88 e Å^−^^3^
0 restraints	Δρ_min_ = −0.93 e Å^−^^3^

### Special details

**Table:**

Geometry. All e.s.d.'s (except the e.s.d. in the dihedral angle between two l.s. planes) are estimated using the full covariance matrix. The cell e.s.d.'s are taken into account individually in the estimation of e.s.d.'s in distances, angles and torsion angles; correlations between e.s.d.'s in cell parameters are only used when they are defined by crystal symmetry. An approximate (isotropic) treatment of cell e.s.d.'s is used for estimating e.s.d.'s involving l.s. planes.
Refinement. Refinement of *F*^2^ against ALL reflections. The weighted *R*-factor *wR* and goodness of fit *S* are based on *F*^2^, conventional *R*-factors *R* are based on *F*, with *F* set to zero for negative *F*^2^. The threshold expression of *F*^2^ > 2σ(*F*^2^) is used only for calculating *R*-factors(gt) *etc*. and is not relevant to the choice of reflections for refinement. *R*-factors based on *F*^2^ are statistically about twice as large as those based on *F*, and *R*- factors based on ALL data will be even larger.

### Fractional atomic coordinates and isotropic or equivalent isotropic displacement parameters (Å^2^)

**Table:**

	*x*	*y*	*z*	*U*_iso_*/*U*_eq_	Occ. (<1)
U1	1.0000	0.0000	1.0000	0.02201 (6)	
O1	1.1934 (3)	0.1523 (3)	1.0498 (3)	0.0299 (4)	
O2	0.7867 (3)	0.0427 (3)	0.7614 (3)	0.0285 (4)	
O3	1.0044 (4)	−0.1789 (3)	0.6978 (3)	0.0372 (5)	
O4	1.1569 (4)	−0.2495 (3)	0.9034 (3)	0.0356 (5)	
O5	1.1808 (5)	−0.3669 (4)	0.6514 (4)	0.0516 (7)	
N1	1.1165 (4)	−0.2698 (4)	0.7464 (4)	0.0316 (5)	
N2	0.7882 (4)	0.2759 (4)	0.7144 (4)	0.0289 (5)	
N3	0.5257 (4)	0.1594 (4)	0.7234 (4)	0.0304 (5)	
C1	0.7028 (4)	0.1579 (4)	0.7359 (4)	0.0237 (5)	
C2	0.7065 (6)	0.4189 (5)	0.6969 (6)	0.0399 (8)	
H2A	0.7498	0.4518	0.6248	0.048*	0.786 (11)
H2B	0.7526	0.5210	0.8181	0.048*	0.786 (11)
H2C	0.6480	0.3901	0.5684	0.048*	0.214 (11)
H2D	0.8101	0.5246	0.7649	0.048*	0.214 (11)
C3	0.4952 (7)	0.3703 (7)	0.6089 (7)	0.0409 (13)	0.786 (11)
H3A	0.4478	0.2848	0.4799	0.049*	0.786 (11)
H3B	0.4444	0.4745	0.6165	0.049*	0.786 (11)
C3B	0.558 (2)	0.457 (2)	0.765 (2)	0.036 (5)	0.214 (11)
H3C	0.6209	0.5204	0.8989	0.044*	0.214 (11)
H3D	0.4844	0.5326	0.7225	0.044*	0.214 (11)
C4	0.4252 (5)	0.2925 (6)	0.6999 (6)	0.0429 (8)	
H4A	0.4452	0.3857	0.8192	0.052*	0.786 (11)
H4B	0.2868	0.2402	0.6258	0.052*	0.786 (11)
H4C	0.3474	0.3176	0.7664	0.052*	0.214 (11)
H4D	0.3375	0.2468	0.5705	0.052*	0.214 (11)
C5	0.9791 (5)	0.2755 (6)	0.7260 (5)	0.0398 (8)	
H5A	1.0191	0.3695	0.7076	0.048*	
H5B	0.9778	0.1639	0.6328	0.048*	
H5C	1.0684	0.2928	0.8452	0.048*	
C6	0.4370 (5)	0.0441 (6)	0.7625 (5)	0.0417 (8)	
H6A	0.3096	0.0628	0.7468	0.050*	
H6B	0.5152	0.0687	0.8871	0.050*	
H6C	0.4266	−0.0769	0.6799	0.050*	

### Atomic displacement parameters (Å^2^)

**Table:**

	*U*^11^	*U*^22^	*U*^33^	*U*^12^	*U*^13^	*U*^23^
U1	0.02333 (8)	0.02264 (8)	0.02564 (8)	0.00962 (5)	0.01238 (6)	0.01470 (6)
O1	0.0291 (10)	0.0298 (10)	0.0382 (12)	0.0080 (8)	0.0153 (9)	0.0220 (9)
O2	0.0297 (11)	0.0333 (11)	0.0295 (11)	0.0151 (9)	0.0139 (9)	0.0198 (9)
O3	0.0454 (14)	0.0466 (13)	0.0370 (12)	0.0299 (11)	0.0269 (11)	0.0261 (11)
O4	0.0427 (13)	0.0364 (12)	0.0313 (11)	0.0211 (10)	0.0169 (10)	0.0185 (10)
O5	0.0618 (18)	0.0590 (17)	0.0440 (15)	0.0396 (15)	0.0344 (14)	0.0222 (13)
N1	0.0307 (13)	0.0339 (13)	0.0309 (13)	0.0141 (11)	0.0148 (11)	0.0151 (11)
N2	0.0274 (12)	0.0338 (13)	0.0355 (13)	0.0140 (10)	0.0169 (11)	0.0221 (11)
N3	0.0238 (12)	0.0409 (14)	0.0348 (14)	0.0131 (11)	0.0153 (11)	0.0227 (12)
C1	0.0234 (13)	0.0308 (13)	0.0215 (12)	0.0125 (11)	0.0107 (11)	0.0155 (11)
C2	0.048 (2)	0.0345 (17)	0.052 (2)	0.0202 (15)	0.0268 (17)	0.0289 (16)
C3	0.046 (3)	0.049 (3)	0.046 (3)	0.032 (2)	0.025 (2)	0.032 (2)
C3B	0.030 (7)	0.027 (7)	0.052 (10)	0.015 (6)	0.023 (7)	0.014 (7)
C4	0.0353 (17)	0.056 (2)	0.054 (2)	0.0285 (17)	0.0270 (17)	0.0332 (19)
C5	0.0278 (15)	0.062 (2)	0.050 (2)	0.0154 (15)	0.0209 (15)	0.0395 (19)
C6	0.0326 (16)	0.055 (2)	0.0447 (19)	0.0059 (15)	0.0193 (15)	0.0282 (17)

### Geometric parameters (Å, °)

**Table:**

U1—O1	1.774 (2)	C2—H2A	0.9900
U1—O1^i^	1.774 (2)	C2—H2B	0.9900
U1—O2^i^	2.363 (2)	C2—H2C	0.9900
U1—O2	2.363 (2)	C2—H2D	0.9900
U1—O4	2.526 (2)	C3—C4	1.521 (6)
U1—O4^i^	2.526 (2)	C3—H3A	0.9900
U1—O3	2.549 (2)	C3—H3B	0.9900
U1—O3^i^	2.549 (2)	C3B—C4	1.494 (16)
O2—C1	1.271 (4)	C3B—H3C	0.9900
O3—N1	1.277 (4)	C3B—H3D	0.9900
O4—N1	1.277 (4)	C4—H4A	0.9900
O5—N1	1.211 (4)	C4—H4B	0.9900
N2—C1	1.348 (4)	C4—H4C	0.9900
N2—C5	1.460 (4)	C4—H4D	0.9900
N2—C2	1.460 (4)	C5—H5A	0.9800
N3—C1	1.350 (4)	C5—H5B	0.9800
N3—C6	1.458 (4)	C5—H5C	0.9800
N3—C4	1.466 (4)	C6—H6A	0.9800
C2—C3	1.484 (6)	C6—H6B	0.9800
C2—C3B	1.514 (15)	C6—H6C	0.9800
			
O1—U1—O1^i^	180	C3—C2—H2A	109.3
O1—U1—O2^i^	87.26 (9)	N2—C2—H2B	109.3
O1^i^—U1—O2^i^	92.74 (9)	C3—C2—H2B	109.3
O1—U1—O2	92.74 (9)	H2A—C2—H2B	108.0
O1^i^—U1—O2	87.26 (9)	N2—C2—H2C	108.9
O2^i^—U1—O2	180	C3B—C2—H2C	108.9
O1—U1—O4	92.17 (9)	N2—C2—H2D	108.9
O1^i^—U1—O4	87.83 (10)	C3B—C2—H2D	108.9
O2^i^—U1—O4	65.69 (8)	H2C—C2—H2D	107.7
O2—U1—O4	114.31 (8)	C2—C3—C4	110.5 (4)
O1—U1—O4^i^	87.83 (10)	C2—C3—H3A	109.6
O1^i^—U1—O4^i^	92.17 (10)	C4—C3—H3A	109.5
O2^i^—U1—O4^i^	114.31 (8)	C2—C3—H3B	109.5
O2—U1—O4^i^	65.69 (8)	C4—C3—H3B	109.6
O4—U1—O4^i^	180	H3A—C3—H3B	108.1
O1—U1—O3	86.07 (10)	C4—C3B—C2	110.3 (9)
O1^i^—U1—O3	93.93 (10)	C4—C3B—H3C	109.6
O2^i^—U1—O3	115.08 (7)	C2—C3B—H3C	109.6
O2—U1—O3	64.92 (7)	C4—C3B—H3D	109.6
O4—U1—O3	50.20 (8)	C2—C3B—H3D	109.6
O4^i^—U1—O3	129.80 (8)	H3C—C3B—H3D	108.1
O1—U1—O3^i^	93.93 (10)	N3—C4—C3B	112.7 (7)
O1^i^—U1—O3^i^	86.07 (10)	N3—C4—C3	111.4 (3)
O2^i^—U1—O3^i^	64.92 (7)	C3B—C4—C3	45.7 (7)
O2—U1—O3^i^	115.08 (7)	N3—C4—H4A	109.4
O4—U1—O3^i^	129.80 (8)	C3—C4—H4A	109.4
O4^i^—U1—O3^i^	50.20 (8)	N3—C4—H4B	109.4
O3—U1—O3^i^	180	C3—C4—H4B	109.4
C1—O2—U1	139.9 (2)	H4A—C4—H4B	108.0
N1—O3—U1	96.59 (18)	N3—C4—H4C	109.1
N1—O4—U1	97.71 (17)	C3B—C4—H4C	109.1
O5—N1—O4	122.5 (3)	N3—C4—H4D	109.1
O5—N1—O3	122.6 (3)	C3B—C4—H4D	109.1
O4—N1—O3	114.9 (3)	H4C—C4—H4D	107.8
C1—N2—C5	120.3 (3)	N2—C5—H5A	109.5
C1—N2—C2	122.8 (3)	N2—C5—H5B	109.5
C5—N2—C2	116.5 (3)	H5A—C5—H5B	109.5
C1—N3—C6	120.6 (3)	N2—C5—H5C	109.5
C1—N3—C4	122.8 (3)	H5A—C5—H5C	109.5
C6—N3—C4	115.9 (3)	H5B—C5—H5C	109.5
O2—C1—N2	119.8 (3)	N3—C6—H6A	109.5
O2—C1—N3	120.7 (3)	N3—C6—H6B	109.5
N2—C1—N3	119.5 (3)	H6A—C6—H6B	109.5
N2—C2—C3	111.4 (3)	N3—C6—H6C	109.5
N2—C2—C3B	113.2 (7)	H6A—C6—H6C	109.5
N2—C2—H2A	109.3	H6B—C6—H6C	109.5

Symmetry codes: (i) −*x*+2, −*y*, −*z*+2.
